# Genomic Architecture and Evolution of the *Cellulose synthase* Gene Superfamily as Revealed by Phylogenomic Analysis

**DOI:** 10.3389/fpls.2022.870818

**Published:** 2022-04-18

**Authors:** Francesco Pancaldi, Eibertus N. van Loo, M. Eric Schranz, Luisa M. Trindade

**Affiliations:** ^1^Plant Breeding, Wageningen University & Research, Wageningen, Netherlands; ^2^Biosystematics group, Wageningen University & Research, Wageningen, Netherlands

**Keywords:** *cellulose synthase* superfamily, CesA, CslD, CslF, evolution, phylogenomics, plant cell walls, synteny analysis

## Abstract

The *Cellulose synthase* superfamily synthesizes cellulose and different hemicellulosic polysaccharides in plant cell walls. While much has been discovered about the evolution and function of these genes, their genomic architecture and relationship with gene (sub-)functionalization and evolution remains unclear. By using 242 genomes covering plant evolution from green algae to eudicots, we performed a large-scale analysis of synteny, phylogenetic, and functional data of the *CesA* superfamily. Results revealed considerable gene copy number variation across species and gene families, and also two patterns – singletons vs. tandem arrays – in chromosomic gene arrangement. Synteny analysis revealed exceptional conservation of gene architecture across species, but also lineage-specific patterns across gene (sub-)families. Synteny patterns correlated with gene sub-functionalization into primary and secondary *CesAs* and distinct CslD functional isoforms. Furthermore, a genomic context shift of a group of cotton secondary *CesAs* was associated with peculiar properties of cotton fiber synthesis. Finally, phylogenetics suggested that primary *CesA* sequences appeared before the secondary *CesAs*, while phylogenomic analyses unveiled the genomic trace of the *CslD* duplication that initiated the *CslF* family. Our results describe in detail the genomic architecture of the *CesA* superfamily in plants, highlighting its crucial relevance for gene diversification and sub-functionalization, and for understanding their evolution.

## Introduction

Plant cell walls (CWs) are versatile structures that surround plant cells and fulfil key plant functions, including providing tensile strength and mediating plant-environment interactions (Sarkar et al., 2009). About 60-90% of CWs’ dry weight is constituted by cellulose and hemicellulosic polymers (Pettolino et al., 2012), synthesized by the enzymes coded by *Cellulose synthase* (*Ces) A* gene superfamily (Richmond and Somerville, 2000). The genes of this superfamily are present in all land plants and globally divided into 12 gene families (Richmond and Somerville, 2000; Yin et al., 2009; Little et al., 2018). Among these families, *CesA* genes were the first discovered members of the *CesA* superfamily and are involved in cellulose synthesis (Pear et al., 1996; Turner and Somerville, 1997; Arioli et al., 1998). These genes are a monophyletic group that the algal ancestors of land plants acquired through horizontal transfer from cyanobacteria that expanded, diversified, and sub-functionalized during plant evolution (Banasiak, 2014, Schwerdt et al., 2015). Specifically, angiosperms typically contain 10-20 *CesA* genes (Richmond and Somerville, 2000; Yin et al., 2009), and different CesA isoforms mediate cellulose synthesis in either primary or secondary cell walls (Kalluri and Joshi, 2004; Burton et al., 2004; Ranik and Myburg, 2006; Wang et al., 2010; Carroll et al., 2012; Liu et al., 2012; Kaur et al., 2016). Moreover, different CesA members assemble into functional hetero-multimeric cellulose synthesis complexes that accomplish the actual cellulose synthesis (Haigler and Roberts, 2019; Carroll et al., 2012). Despite the recent advances in the knowledge of *CesA* biology, neither the evolutionary trajectories that led to the current *CesA* diversity nor the genomic architecture of this family and its relationship with CW biology are fully understood (Little et al., 2018).

The other *CesA* superfamily genes besides the *CesA* family are termed *Cellulose synthase-like* (*Csl*) genes. *Csl* genes participate in the synthesis of different hemicellulosic polysaccharides (Richmond and Somerville, 2000; Schwerdt et al., 2015, Little et al., 2018) and include 11 families, termed *CslA* to *CslM* (Little et al., 2018). The *CslB*/*D*/*E*/*F*/*G*/*H*/*J*/*M* families belong to the same monophyletic lineage of the *CesA* genes (Banasiak, 2014; Little et al., 2018), while the *CslA*/*C*/*K* families form a different clade that originated through an independent endosymbiosis in green algae (Yin et al., 2009; Banasiak, 2014, Little et al., 2018). Within the *CslB*/*D*/*E*/*F*/*G*/*H*/*J*/*M* families, the *CslD* and *CslF* clades are phylogenetically closer to *CesA* genes than the others (Yin et al., 2009; Schwerdt et al., 2015). Specifically, *CslDs* form a sister clade *CesAs*, while *CslFs* form a Poaceae-specific sister clade of *CslDs*. While phylogenetic analyses suggest that *CslF* genes originated from *CslD* duplication (Yin et al., 2009; Schwerdt et al., 2015; Little et al., 2018), it is unclear if such duplication took place after the divergence of the *CslD* clade, or in an ancestral clade of both the families (Little et al., 2018). Similarly, the *CslB*/*E*/*G*/*H*/*J*/*M* families diverged independently of the *CslD* and *CslF* genes from the *CesA* family (Yin et al., 2009; Little et al., 2018), but the timing and modes of their evolution is largely undefined (Banasiak, 2014). Furthermore, the role of the genomic architecture of genes in the evolutionary trajectories of all these families is unclear.

Knowledge gaps are also currently open regarding the function of the *Csl* families. In this respect, the *CslF*, *CslH*, and *CslJ* families are known to be involved in the synthesis of (1,3;1,4)-β-glucans (or mixed-linkage glucans), a group of polymers mainly found in Poaceae (grass) CWs that consists of (1,3;1,4)-β linked glucosyl residues (Burton et al., 2006; Doblin et al., 2009; Little et al., 2018). While the involvement of the *CslF*, *CslH*, and *CslJ* families in the synthesis of mixed-linkage glucans has been widely established over the last decades, two members of the barley *CslF* family – *HvCslF3* and *HvCslF10* – have recently been shown to synthesize (1,4)-β-linked glucoxylans (Little et al., 2019). This finding can challenge the concept that *CslF*, *CslH*, and *CslJ* families synthesize a single type of polysaccharide. Nevertheless, the *CslF*, *CslH*, and *CslJ* families remain the best studied *Csl* families, while the other *Csl* genes are much less characterized. Some *CslA* genes synthesize mannans and glucomannans (Dhugga et al., 2004; Liepman et al., 2005), while certain CslC isoforms mediate xyloglucan biosynthesis (Cocuron et al., 2007; Kim et al., 2020). However, the size and diversity of these families suggests that they could also synthesize other hemicellulosic polysaccharides or different forms of mannans and xyloglucans (Liepman and Cavalier, 2012). *CslD* genes are believed to synthesize the non-crystalline single chains of cellulose in root hairs and pollen tubes (Doblin et al., 2001; Kim et al., 2007; Bernal et al., 2008), but their involvement in mannan synthesis has also been proposed (Verhertbruggen et al., 2011). In addition, *CslD* mutations affect pollen development (Bernal et al., 2008), root morphology (Wang et al., 2001; Kim et al., 2007; Bernal et al., 2008; Hu et al., 2018), and vegetative organ size (Li et al., 2009; Luan et al., 2011; Hunter et al., 2012; Li et al., 2018) in several plants, but the molecular basis of these effects is unclear. Finally, the function of the evolutionarily-related *CslB*/*E*/*G*/*M* families is currently unknown (Little et al., 2018). Moreover, as for the *CesA* genes, the role of the genetic architecture of the *Csl* families in determining gene function has not yet been investigated.

The study of the genomic architecture of the *CesA* superfamily in plants and its relationship with the evolution and function of these genes is the focus of this research. To this aim, we performed a combined phylogenetic and synteny analysis (phylogenomic analysis) of the *CesA* superfamily genes from 242 species covering plant evolution from green algae to eudicots. Large-scale phylogenomic analyses are a rather novel approach, but have turned powerful in studying the genetics and the evolution of complex gene families (Zhao et al., 2017; Kerstens et al., 2020). Moreover, if coupled with relevant phenotypic and functional data, these analyses can reveal relationships between the genomic architecture of target gene families and phenotypic adaptations of plants (Zhao et al., 2017; Kerstens et al., 2020). Large-scale phylogenomic analyses have become feasible thanks to the increasing availability of sequenced plant genomes and the development of bioinformatic tools for their analysis, like network approaches for large-scale synteny computation (Zhao and Schranz, 2017). The application of these tools to the *CesA* superfamily highlighted interesting patterns in the genomic arrangement of these genes across plants and relevant associations between phylogenomic patterns and key events in the evolution and sub-functionalization of different gene families, including *CesA*, *CslD*, and *CslF* genes. This also led to the formulation of novel hypotheses on the evolution of different *CesA* and *Csl* families.

## Materials and Methods

### Genomic Data Sources

All the plant genomes (*sensu lato*) sequenced and published by 2018 and available with a scaffold-level assembly were searched in online databases (Supplementary Table 1). For each genome, a general feature format (GFF)/browser extensible data (BED) file of gene positions and a protein FASTA file reporting the main protein coded by each gene were retrieved. Moreover, genomes were analysed for completeness and fragmentation by using the BUSCO Viridiplantae gene set (Seppey et al., 2019) and by assessing the number of scaffolds and the N50 statistics. In total, 242 genomes from 212 species were collected (Supplementary Table 1). For each species with an available genome, information on its ploidy level and the number of genome duplications were searched online and on scientific literature [see especially Van de Peer et al. (2017) for information about genome duplications].

### Identification of CesA and Csl Genes

A group of 445 protein sequences of known *CesA*/*Csl* genes from 13 species spanning plant diversity were retrieved from literature and used as BLAST queries (Altschul et al., 1990) against the 242 proteomes of the study (Supplementary Table 2). In parallel, all the proteins of the 242 genomes were screened for the characteristic PFAM domains of the *CesA* superfamily – PF03553 and PF00535 (Little et al., 2018) – using HMMER (Eddy, 2009; El-Gebali et al., 2019). The outputs of the BLAST and HMMER searches were merged and filtered to exclude partial sequences not starting with Methionine and/or shorter than the residue length of the PFAM domains annotated onto them (total residues spanned by each PFAM; 170 AAs for PF00535 and 722 AAs for PF03552). The remaining sequences were assigned to *specific CesA*/*Csl* families through a second BLAST against the *CesA*/*Csl* genes from the initial 13 species for which a *specific CesA*/*Csl* function was and by plotting them in a phylogenetic tree to identify wrongly annotated and spurious sequences (R, custom script).

### Identification of Primary and Secondary CesA Genes

For the phylogenomic analysis of primary and secondary *CesA* genes, a set of 49 experimentally validated primary and secondary *CesA* sequences was retrieved from literature (Supplementary Table 3) and used in a BLAST search (Altschul et al., 1990) against all the *CesA* genes found in the genomes of the study. BLAST results were used to preliminarily categorize all *CesA* genes as primary or secondary *CesAs*. This initial assignment was refined by checking the simultaneous presence of two motifs (CQIC and SVICEXWFA) previously shown to characterize only primary *CesA* proteins in a wide range of plants (Kaur et al., 2016). Moreover, the position of each *CesA* gene found in the BLAST search, relative to the clades where the initial 49 primary and secondary *CesAs* were placed, was manually inspected in each phylogenetic tree of this study to further help the categorization of primary and secondary *CesA* genes following the BLAST search.

### Synteny Network Construction

The synteny network of the *CesA* superfamily was built by following the methodology of Zhao and Schranz (2017). In brief, we used Diamond (Buchfink et al., 2015) to perform BLAST-like alignments of all the proteins of each genome against all the other proteins of that genome and all the proteins of every other genome (Evalue = 1E–3). In this way, we identified homologous genes between different species. Subsequently, MCScanX (Wang et al., 2012) was run with default parameters (except -s – the number of colinear genes to claim a syntenic block – set to 3) to detect gene synteny (i.e., conserved gene order across multiple genomes). The results of MCScanX were organized in a synteny network, in which each node is a gene and edges represent syntenic connections between genes. The set of edges in which at least one node was a *CesA*/*Csl* gene was extracted from the overall synteny network and represents the *CesA*/*Csl* synteny network (Supplementary Table 4).

### Analysis of Syntenic Communities

The R package igraph and the Multi-level algorithm (Yang et al., 2016) were used to detect syntenic communities within the *CesA*/*Csl* network (i.e., groups of *CesA*/*Csl* genes displaying a higher degree of synteny with each other than with the rest of the network) formed by at least four nodes. Communities were profiled to assess the type(s) of *CesA*/*Csl* families and species included in each of them, and the gene copy number of each *CesA*/*Csl*-taxa combination was found. Finally, communities were hierarchically clustered based on *CesA*/*Csl* copy number per species (R, hclust function, ward.2 algorithm).

### Multiple Sequence Alignment and Phylogenetic Analysis

Phylogenetic trees were built for relevant groups of *CesA*/*Csl* genes. For each tree, full-length CesA/Csl protein sequences were aligned with MAFFT v7.453 (FFT-NS-2 algorithm) (Katoh and Standley, 2013), with default parameters except gap opening penalty, at 1.0. MAFFT alignments were set and trimmed with TrimAl v1.2 (Capella-Gutiérrez et al., 2009), with manual optimization of the -gt and -cons flags to obtain alignments lengths similar to the median lengths of the initial proteins included in each tree. Phylogenetic trees were built from trimmed alignments using RAxML v8.2.9 (PROTCATBLOSUM62 substitution matrix; 100 bootstraps) (Stamatakis, 2014), and plotted and annotated using iTOL (Letunic and Bork, 2019).

### Statistical Analyses

All the statistical analyses (*t*-tests, ANOVAs, LSDs, correlations) described in this research were performed with the Statistical Package for the Social Sciences (SPSS; IBM Corp., Armonk, NY, United States; Version 26.0).

## Results

### Genomic Architecture of the CesA Superfamily

#### Gene Copy Number

We used 242 plant genomes, covering plant evolution from green algae to eudicots (Supplementary Table 1), to perform an extensive genomic and synteny analysis of the *CesA* superfamily in plants, of which the first step was the study of its genomic architecture. The 222 plant genomes with a BUSCO representation ≥75% contained 7,997 *CesA* superfamily genes, with an average of 36 members per species (CV = 48.6%; Supplementary Table 5; CV: Coefficient of Variation). The copy number of *CesA*/*Csl* genes correlates with both the ploidy level and the number of genome duplications of each species (ρ = 0.57 and ρ = 0.55, respectively; *p* < 0.001 for both; Supplementary Figure 1). Moreover, the *CesA* superfamily size increased considerably during plant evolution, with an acceleration at the rise of angiosperms. In fact, while charophytes, bryophytes, lycophytes, ferns, and gymnosperms contain a relatively similar number of *CesA*/*Csl* genes (∼18 per species, CV = 36.9%), this figure doubles in angiosperms despite similar inter-species variability (∼40 *CesA*/*Csl* genes per species, CV = 35.9%, *p* < .001; Supplementary Figure 2).

*Cellulose synthase A* is by far the most abundant *CesA*/*Csl* family, with 12 genes per species on average (CV = 43.1%; Supplementary Figure 3). This is roughly two times the size of the *CslD*, *CslA*, and *CslC* families (*p* < 0.001), which all display ∼6 genes per species. These three families are in turn significantly larger than the *CslB*, *CslE*, *CslG*, and *CslM* families (1-3 genes per species each; *p* < 0.001). Finally, the monocot-specific *CslF* and *CslH* families typically display 7 and 1 genes per species on average, respectively. Overall, the copy number of all the *CesA*/*Csl* families varies extensively across plant families, with standard deviations often equal to the mean size of gene families across species.

#### Gene Distribution Along Genomes

The relative positions of the *CesA/Csl* genes within each genome of the study were also assessed, highlighting two main patterns (Supplementary Tables 6–8). On the one hand, the *CesA*, *CslA*, *CslC*, and *CslD* families display a scattered genomic distribution among multiple singleton loci. On the other hand, the *CslB*, *CslE*, *CslG*, and *CslM* clades are usually organized in tandem arrays of 2–4 genes. These arrays were detected in both monocots and dicots, and synteny analysis showed their extensive conservation across species (see section “Gene Synteny”). A large conserved gene array was also found for the *CslF* family in all the Poaceae species evaluated [as previously reported by Schwerdt et al. (2015) for rice, sorghum, Brachypodium and barley only]. However, we also found 20–30% of the *CslF* genes of each Poaceae species organized as conserved singletons or tandems at separated loci. Interestingly, this second group of *CslF* genes was syntenic with *CslD* members of several eudicot species (see section “Gene synteny” and “Synteny Reveals the Genomic Trace of CslF Evolution”). Finally, the *CslH* and *CslJ* families displayed both configurations – tandem arrays and singleton loci. However, these families are present at too low frequencies and in too few species to allow generalizations.

#### Gene Synteny

**FIGURE 1:**
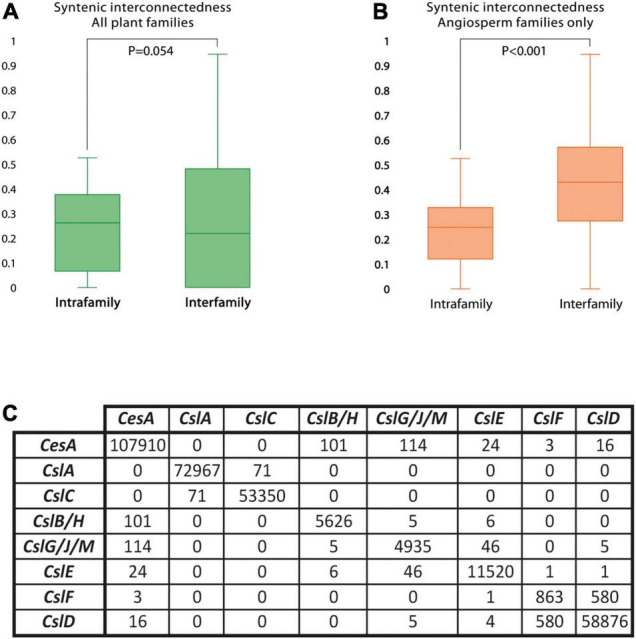
The degree and structure of *Cellulose synthase/Cellulose synthase-like* (*CesA*/*Csl)* gene synteny. Synteny is, overall, high and extensive across plant families, but arranged differently across gene families. **(A)** Distribution of the coefficients representing the average number of syntenic connections per gene in the *CesA*/*Csl* synteny network with other genes from the same (intrafamily) or from different (interfamily) plant species. Coefficients were calculated using data from all the plant families included in the analysis. **(B)** Distribution of the coefficients representing the average number of syntenic connections per gene in the *CesA*/*Csl* synteny network with other genes from the same (intrafamily) or from different (interfamily) plant species. Coefficients were calculated using data from only angiosperm families. **(C)** Total number of syntenic connections detected between gene families within the *CesA* superfamily.

The syntenic conservation of the *CesA* superfamily was also assessed in detail by using network synteny analysis. Results indicated that *CesA* superfamily genes are highly syntenic across diverse plants, more than what observed for other plant gene families, including regulatory genes. In fact, of the 7,005 *CesA* superfamily genes from the 193 genomes with a BUSCO representation ≥75% and at least five genes per scaffold on average, 6,262 (89%) were included into the *CesA*/*Csl* synteny network (Supplementary Table 9). This is a remarkably high proportion, 9 and 24% higher than what is found in comparable studies on the *MADS-box* and the *APETALA2* gene families, respectively (Zhao et al., 2017; Kerstens et al., 2020). Furthermore, the synteny of the *CesA*/*Csl* genes is dense and extensive across diverse plant species and families. In fact, each *CesA*/*Csl* gene is syntenic to another 69 homologs from 46 different plant species on average. Moreover, the degree of gene synteny does not significantly differ between intra- and inter-family syntenic comparisons (*p* = 0.054, Figure 1A). Remarkably, for angiosperms only, syntenic conservation is even higher between than within plant families (*p* < 0.001, Figure 1B) and crosses the boundaries of monocots and dicots. This trend is clearly divergent from the patterns commonly observed for plant genes (Zhao and Schranz, 2019).

**FIGURE 2:**
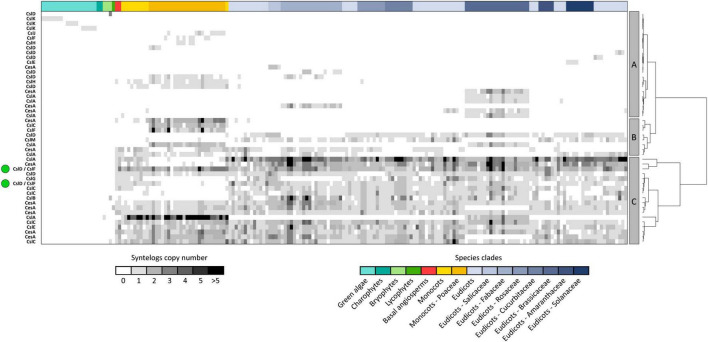
The 48 syntenic communities detected within the *CesA/Csl* synteny network. Syntenic communities are groups of genes displaying higher degrees of synteny with each other than with the rest of the network. Therefore, they constitute conserved architectural configurations of genes across genomes of different species. The figure highlights that *CesA/Csl* communities typically harbour genes belonging to the same *CesA/Csl* family, except for two communities grouping *CslF* and *CslD* genes (green dots on the left of the figure). *CesA/Csl* syntenic communities are also divided into three main clusters based on the spread of their genes across the plant kingdom (**A–C** panels and dendrogram at the right of the figure). In the figure, rows represent communities while columns represent species. Cells are coloured according to the number of syntenic genes harboured by each community and each species within communities. Row headers on the left side indicate the predominant gene family harboured by each community (>90% of the community members).

While synteny is dense and extensive across different plants, the same does not hold across different *CesA*/*Csl* families, which are organized in separate conserved genomic contexts. Accordingly, 99% of syntenic connections within the *CesA*/*Csl* synteny network takes place between genes from the same *CesA*/*Csl* family (Figure 1C). Moreover, each of the 48 syntenic communities identified by decomposing the *CesA*/*Csl* synteny network in groups of highly syntenic genes typically contains genes from only one *CesA*/*Csl* family (Figure 2). Furthermore, multiple communities were detected for most of the *CesA*/*Csl* families, revealing distinct conserved genomic contexts even at the level of gene subclades within *CesA*/*Csl* families (Figure 2). In this respect, the marked differentiation in the genomic organization of the *CslA* genes between monocots (especially Poaceae) and dicots is noteworthy (group B and parts of Groups A and C, Figure 2). Moreover, syntenic communities for specific plant families were detected for some *CesA* and *CslA* genes within Salicaceae, Fabaceae, and Brassicaceae (Group A of Figure 2). Next to these examples of intra-family sub-organization of gene architecture, we also found communities that cross the boundaries of single *CesA*/*Csl* gene families (Group C of Figure 2). Specifically, two communities, including 580 syntenic connections between *CslF* and *CslD* genes, appeared particularly relevant as they grouped ∼40% of all the syntenic connections involving *CslF* genes (Figure 2).

### Relationship Between Genomic Architecture and Gene Properties

#### Distinct Phylogenomic Features for Distinct *CesA* Isoforms

**FIGURE 3:**
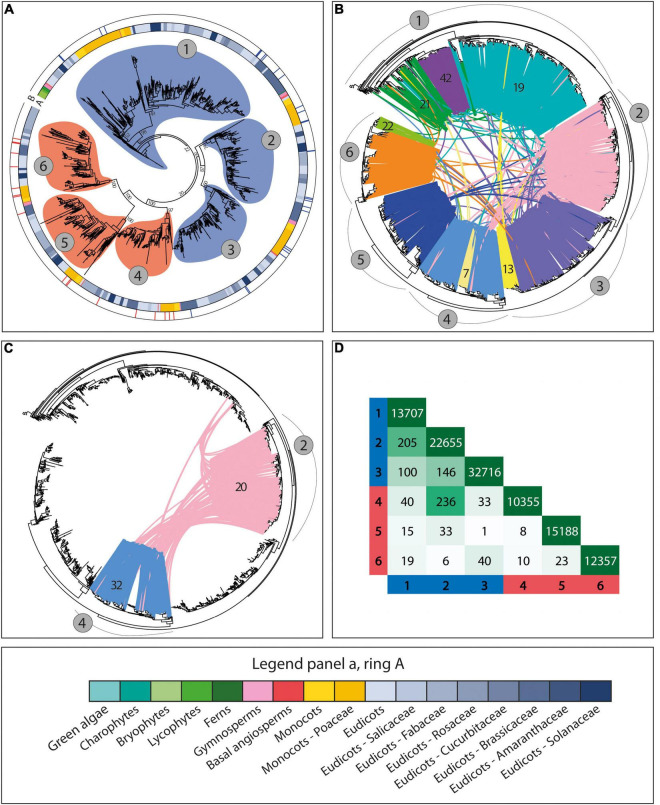
The phylogenomic structure of the *CesA* family. **(A)** The phylogenetic tree of all the *CesA* genes from the 193 genomes with a BUSCO representation ≥75% and at least five genes per scaffold on average. The tree highlights the subdivision of the *CesA* sequences into six main phylogenetic groups corresponding to known primary and secondary cell wall *CesA* isoforms from different species. Rings A and B, respectively, represent the species taxonomy of the different genes (legend at the bottom of the figure) and the tree position of 49 experimentally validated primary (blue) and secondary (red) cell wall *CesA* genes. Circles around tree branches highlight the six major *CesA* clades detected in the tree, divided into three primary (blue) and three secondary (red) cell wall *CesA* clades. **(B)** The phylogenetic tree of all the *CesA* genes as in panel A, with highlighted the syntenic connections between genes and the different syntenic communities detected. Lines connecting tree leaves indicate syntenic connections between genes, with different colours highlighting different syntenic communities. Numbers in grey circles refer to gene clades as in panel A, while numbers on coloured syntenic connections indicate community numbers as referred in the article. The figure shows that the six major *CesA* clades of panel A are arranged into distinct conserved architectural genomic configurations (or genomic contexts). **(C)** Highlight of the syntenic connections between clades 2 (pink; primary cell wall *CesA* genes) and 4 (blue; secondary cell wall *CesA* genes). The pink links between the two phylogenetic clades indicate secondary cell wall *CesA* genes placed in genomic contexts typical of primary cell wall *CesAs*. These sequences come mostly from the Malvaceae family, and specifically from the three cotton species therein: *G. hirsutum*, *G. arboreum*, and *G. raimondii*. In *G. hirsutum*, the species involved in these connections were shown to be at the basis of the massive fibre deposition observed in cotton. **(D)** Total number of syntenic connections detected between the six clades of *CesA* genes as represented in the tree of panel A. Row and column headers refer to the six clades of the tree in panel A.

To further characterize the genomic patterns described above and to study their relevance for the evolution and sub-functionalization of the *CesA*/*Csl* genes, we investigated how such patterns relate to phylogenetic and functional gene data. For the *CesA* family, our results revealed striking correspondence between the genomic organization of genes, the syntenic conservation of gene architecture, the phylogenetic relationships between genes, and the diversification of genes into distinct isoforms with different functional properties. Specifically, the *CesA* phylogeny was divided into six main clades supported by moderate-to-high bootstraps, corresponding to the six main groups of CesA isoforms known in *Arabidopsis thaliana* and *Oryza sativa* (Figures 3A, 5). These six clades are grouped into two separated super-groups of three clades each, corresponding to the subdivision into primary and secondary CW *CesA* genes (Figures 3A, 5 and Supplementary Table 3). Interestingly, each of the six phylogenetic clades is differentially organized and differentially conserved at the genomic level by spanning only one of the six largest *CesA* syntenic communities found in the *CesA*/*Csl* synteny network (Figures 3B,D). Overall, these six clades and communities span 84% of the syntenic *CesA* genes and cover 93% of the angiosperm families included in the synteny analysis. The correspondence between *CesA* phylogeny and synteny is therefore striking, with only one major exception found for clade 1 of Figures 3A,B, which is subdivided into a main syntenic community largely dicot-specific (community 19 of Figure 3B) and other two smaller communities, of which one is specific to monocots (community 42 of Figure 3B) and one is a group of diverse *CesA* members (community 21 of Figure 3B). Besides this clade, only few other minor groups of genes specific to certain plant families deviate from the common phylogenetic and syntenic structure described above by being organized in distinct genomic contexts. Examples are a Fabaceae-specific community from clade 6 of Figure 3B (community 22 of Figure 3B), a Brassicaceae-specific community from clade 3 of Figure 3B (community 13 of Figure 3B), and a community from clade 4 of Figure 3B specific to Brassicaceae and Malvaceae (community 7 of Figure 3B).

#### A Genomic Context Shift Associated to Specific *CesA* Properties in Cotton

Overall, the six different *CesA* phylogenomic clades are genomically independent from each other, with seldom inter-clade syntenic connections (Figures 3B,D). An exception is represented by the 236 syntenic links between clades 2 (primary CW *CesAs*) and 4 (secondary CW *CesAs*) of Figures 3B,C. A minor part of these connections is spread across different angiosperm families and can be regarded as background noise of the synteny analysis. However, 194 connections specifically involve the Malvaceae family and three cotton species therein: *G. hirsutum*, *G. arboreum*, and *G. raimondii*. These connections mainly involve three primary and five secondary cotton *CesA* genes from branches 2 and 4, respectively, which are syntenic to a total of 104 *CesA* genes from 78 different angiosperm genomes placed in opposite phylogenetic groups (branch 4 for the cotton *CesAs* from branch 2, and branch 2 for the cotton *CesAs* on branch 4) (Figure 3C). This suggests that the cotton secondary CW *CesA* genes involved in these connections changed their genomic position relative to the other angiosperm genes from the same phylogenetic group, ending up in a genomic context typical of primary CW *CesAs* (clade 2 of Figures 3B,C).

Remarkably, the *CesA* genes of *G. hirsutum* involved in the genomic inversion turned out to be associated with distinct functional properties at the basis of the massive fibre production of cotton. These are three different *G. hirsutum CesA* members: an already known *CesA7*, an already known *CesA8*, and a previously uncharacterized homolog of *GhCesA8* (99.6% sequence identity) (Supplementary Tables 10, 11). Interestingly, these three isoforms have been proved to assemble together into functional rosettes for cellulose deposition in *G. hirsutum* fibre cells, with *CesA8* specifically acting as enhancer for massive fibre deposition (Li et al., 2016, 2013). In addition, the biological mechanism of fibre synthesis involving *G. hirsutum CesA7* and *CesA8* appears conserved across different cotton species, including the ones included in our analysis and showing the same genomic inversion (Li et al., 2013). Accordingly, all the secondary CW CesA isoforms from *G. arboreum* and *G. raimondii* that are involved in the genomic context shift share 95.6% sequence identity with the *G. hirsutum CesA7* and *CesA8* for which functional data were available in scientific literature (Supplementary Table 11). Overall, these results suggest that the genomic positioning and organization of *CesA* genes might be critical for determining gene function, representing an important factor at the basis of cotton fibre deposition.

#### Differential Genomic Contexts for Different Gene Functions in the *CslD* Family

**FIGURE 4:**
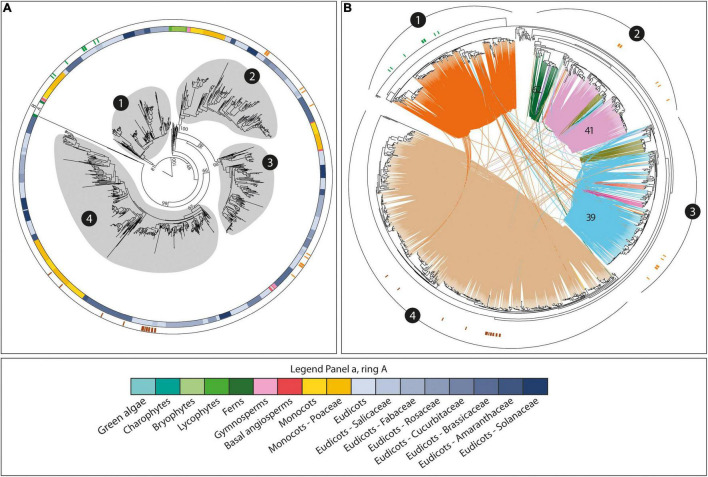
The phylogenomic structure of the *CslD* family. The trees highlight the association between the phylogenetic and the conserved (syntenic) architectural structure of the family, and also the functional diversity of the *CslD* genes. **(A)** The phylogenetic tree of all the *CslD* genes from the 193 genomes with a BUSCO representation ≥75% and at least five genes per scaffold on average. Ring A represents the species taxonomy of the different genes (legend at the bottom of the figure). Ring B represents the tree position of several *CslD* genes known to be involved in the determination of vegetative organ size (green), pollen development (orange), or root hair formation (brown). Circles around tree branches highlight the four major *CslD* clades detected in the tree. Bootstrap values supporting clades subdivisions are reported. **(B)** Syntenic structure of the four *CslD* phylogenetic clades. Lines connecting tree leaves indicate syntenic connections between genes, with different colours highlighting different syntenic communities. Numbers in black circles refer to the four clades of panel A, while numbers on syntenic connections indicate community numbers as referred in the article.

Striking correspondence between genomic organization, syntenic conservation, phylogenetic relationships, and functional diversification was also found within the *CslD* family. This family is structured into four main phylogenetic clades (Figure 4A). In addition, previous studies demonstrated the involvement of different *CslD* members into three main plant processes – pollen development, determination of vegetative organ size, and root hair formation – across several species (Wang et al., 2001; Kim et al., 2007; Bernal et al., 2008; Li et al., 2009; Luan et al., 2011; Hunter et al., 2012; Hu et al., 2018; Li et al., 2018). Strikingly, the functional subdivision of the *CslD* members overlapped with the main phylogenetic *CslD* clades (Figure 4). In fact, known *CslD* genes involved in determination of vegetative organ size and root hair formation were only found within clades 1 and 4, respectively, while *CslD* members known to be involved in pollen development were only observed in clades 2 and 3 (Figure 4). In turn, the different phylogenetic and functional *CslD* groups corresponded to distinct and highly conserved *CslD* syntenic communities, highlighting their independent organization and conservation across plants (Figure 4B). However, while one syntenic community was found for each of the phylogenetic clades grouping *CslD* genes involved in organ size determination and root hair formation, respectively, multiple communities specific to different taxonomic groups were detected within the *CslD* branch associated to pollen development (Figure 4). Of these, one is broad and spans both monocot and dicot species (community 39), one is large but restricted to dicots (community 41), and four are minor and restricted to specific taxonomic clades.

While the subdivision of *CslD* genes described above is shared by all angiosperms, the same does not hold for earlier land plants. In fact, *CslD* members from bryophytes and lycophytes are found in only a single phylogenetic group placed between the *CslD* branches associated to organ size determination and pollen development. Moreover, fern *CslDs* are divided into two groups that are closest to angiosperm *CslD* members involved in organ size determination and root hair formation, respectively. Finally, *CslD* genes from bryophytes, lycophytes, ferns, and gymnosperms do not display any syntenic connection with the angiosperm ones.

#### Phylogenomic Patterns in Other *Csl* Families

Commonalities between phylogenetic, syntenic, and functional patterns were also found in other *Csl* families. However, for the evolutionarily close *CslB*/*H*/*G*/*J*/*M*/*E* families, such commonalities are observed for whole families rather than gene subclades within families (Supplementary Figure 4), with deviations observed only in a *CslM* syntenic community specific to Fabaceae (community 23 of Supplementary Figure 4) and a small *CslE* community with only *Capsicum* genes (community 17 of Supplementary Figure 4). Finally, *CslA* and *CslC* genes also display syntenic suborganization of their phylogenies (Supplementary Figure 5). However, the functional information available for these families are not abundant and do not reveal any clear correspondence with the phylogenetic and/or syntenic structure observed.

### Evolutionary Dynamics of Specific Gene Families

#### Evolution of the *CesA* Genes

**FIGURE 5:**
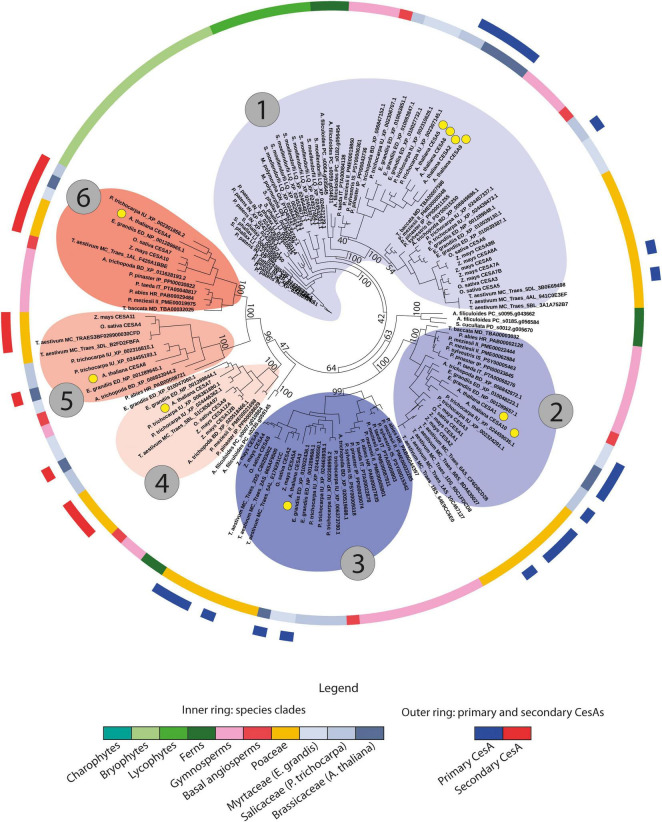
Phylogenetic tree of a subset of 151 *CesA* genes including sequences from model plant species, from species where primary and secondary cell wall *CesA* genes have been experimentally validated (Supplementary Table 3), along with all gymnosperm, fern, lycophyte, bryophyte, and charophyte *CesA* sequences. The tree shows that early-diverging plant sequences are only occurring closest to primary cell wall *CesA* branches (especially clade 1). Moreover, it highlights the subdivision of the *CesA* sequences into the six main phylogenetic groups corresponding to known primary and secondary cell wall CesA isoforms from different species. The inner ring indicates taxonomy, while the outer ring highlights the position of experimentally validated primary and secondary cell wall *CesA* genes (see legend at the bottom of the figure). Coloured clades around branches indicate the six main *CesA* clades, numbered in the same way as in Figure 3. Blue clades indicate primary cell wall *CesA* genes, while red clades indicate secondary cell wall *CesA* sequences. Bootstraps supporting clade subdivisions are reported. Yellow dots indicate the position of the 10 *A. thaliana CesA* genes.

Phylogenetic and synteny data were used to also study the evolution of the main *CesA*/*Csl* families. For the *CesA* genes, the taxonomic profiling of the six main *CesA* clades (see section “Distinct Phylogenomic Features for Distinct CesA Isoforms”) revealed that *CesA* sequences of bryophytes and lycophytes are phylogenetically closest to the primary CW *CesA* branches, and specifically to the primary CW *CesA* genes homolog to the redundant Arabidopsis *CesA2/5/6/9* and the *O. sativa CesA3/5/6* (clade 1 of Figure 5). This observation suggests that primary CW *CesAs* are the oldest *CesA* sequences of land plants, and that early land plants likely only had primary CW *CesA* (-like) genes. The positioning of fern *CesAs* suggests that the diversification of *CesA* genes toward the six main angiosperm clades described in section “Distinct Phylogenomic Features for Distinct CesA Isoforms” started sometime during the late lycophyte or early fern evolution. In fact, while a group of fern *CesAs* is also positioned close to the bryophyte and lycophyte sequences of clade 1 of Figure 5, another group of fern genes precede the split of the other two clades of angiosperm primary CW *CesAs* (clades 2 and 3 of Figure 5). Moreover, a third group of fern *CesAs* is closest to the three groups of angiosperms secondary CW *CesAs* (clades 4, 5, and 6 of Figure 5), just preceding their diversification. Therefore, *CesAs* diversification toward the different phylogenomic groups observed in higher plants significantly progressed during fern evolution. However, gymnosperms are the first group of plants whose *CesA* sequences are found in all the six *CesA* clades genomically conserved across all the angiosperms. To conclude, data on gene copy-number within each of the six *CesA* clades also support the evolutionary model discussed. In fact, the early-diverging clade 1 contains by far the most *CesA* sequences in both Figures 3A, 5 (771 sequences in the full *CesA* tree of Figure 3A), followed by clades 2 and 3 of primary CW *CesAs* (394 genes on average, CV = 4.3%), and finally by the three secondary CW *CesA* clades (273 genes each on average, CV = 6.3%). These data, together with the presence of redundant Arabidopsis *CesA* copies within two primary CW *CesA* clades (1 and 2 of Figure 5), agree with the view that oldest *CesA* families evolved first into multiple isoforms, of which some underwent full sub-functionalization within the cellulose synthesis machinery [a model known as constructive neutral evolution, recently advanced by Haigler and Roberts (2019) for the *CesA* genes – see discussion].

#### Synteny Reveals the Genomic Trace of *CslF* Evolution

**FIGURE 6:**
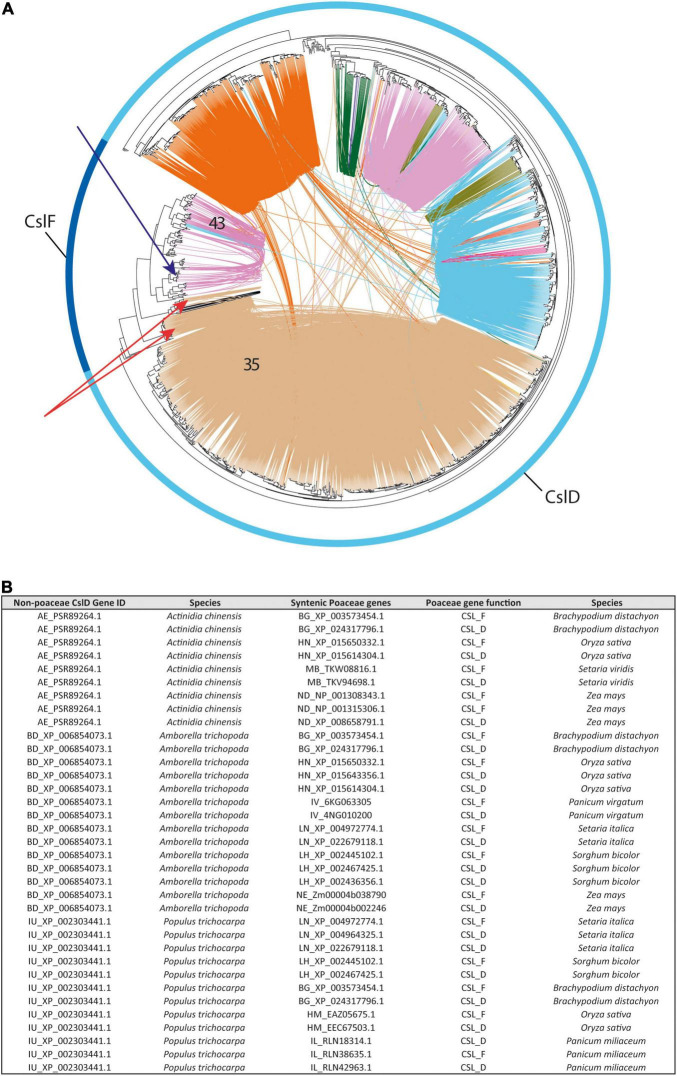
The 1:2 syntenic connections between a group of angiosperm *CslD* genes and a class of Poaceae *CslD* and *CslF* genes. **(A)** Phylogenetic tree of all the *CslD* and *CslF* genes from the 193 genomes with a BUSCO representation ≥75% and at least five genes per scaffold on average. The outer ring highlights *CslD* and *CslF* tree branches. Red arrows indicate the *CslF* genes syntenic to *CslD* isoforms involved in root morphology (same syntenic community as in Figure 4). Blue arrow indicates the positioning of *HvCslF3* and *HvCslF10*, the two *CslF* genes responsible for the synthesis of 1,4-β-linked glucoxylan in barley. **(B)** Table reporting examples of syntenic connections between three *CslD* genes from three different angiosperm species and both *CslF* and *CslD* genes across different Poaceae species.

In the *CslF* family, our phylogenomic analysis detected 580 syntenic connections between the *CslF* genes organized as singletons or tandems in the Poaceae genomes (see section “Gene Distribution Along Genomes”) and a subset of *CslD* members, all included within the same syntenic community (community 35 in Figure 6 and green dots in Figure 2). Interestingly, 327 of these connections involve *CslD* genes from 53 different eudicot species (27 different plant families) that display simultaneous synteny with a grass *CslD* gene and a grass *CslF* member in 17 of the 21 Poaceae genomes of our study (Figure 6B). The frequency of these connections is negatively correlated with the evolutionary distance of the species harbouring “seed” *CslD* sequences from Poaceae (*r* = –0.57). Moreover, the “seed” *CslD* genes all belong to the *CslD* phylogenomic clade grouping *CslD* genes involved in root hair formation (see section “Gene Distribution Along Genomes”). All together, these observations reveal the genomic trace of the *CslF* origin in grasses. On the one hand, they confirm that the *CslF* genes originated through the duplication of some *CslD* members (Yin et al., 2009; Schwerdt et al., 2015; Little et al., 2018). On the other hand, they show that the *CslF* family is nested within the *CslD* one (and specifically within the *CslD* clade involved in root hair formation). This in turn proves that the *CslF* genes took origin when the *CslD* family was already formed, thanks to a relaxed evolutionary pressure on duplicated *CslD* members in grasses. To conclude, it is noteworthy that no synteny was detected between the *CslF* genes syntenic to *CslDs* (red arrows in Figure 6) and the other *CslF* members included in community 43 (Figure 6), which form the conserved *CslF* genomic array (see section 2.1.2). The fact that only some *CslF* genes – corresponding to one of the two architectural configurations of this family – display synteny with *CslD* members questions whether the *CslF* family originated through a single *CslD* duplication, or if the family underwent different rounds of expansion in Poaceae. In this respect, the phylogenomic positioning of the barley *CslF* members recently shown to synthesize (1,4)-β-linked glucoxylans was checked in the tree of Figure 6A (blue arrow). Interestingly, both *HvCslF3* and *HvCslF10* are positioned within the phylogenetic clade spanned by community 43, which does not display synteny with *CslD* members.

## Discussion

### The Genomic Architecture and the Evolution of the CesA Superfamily

In section “Genomic Data Sources”, we showed that the genomic architecture of the *CesA* superfamily varies considerably in terms of gene copy number, gene distribution along genomes, and gene synteny across different plant and gene families. These results provide insight into the timing and modes of *CesA* superfamily evolution. Specifically, a key finding is that the *CesA*/*Csl* copy number variation correlates with the evolutionary timing of the *CesA* superfamily. Accordingly, we found that *CesA* is the largest *CesA*/*Csl* family, followed by the *CslD/F*, *CslA*, and *CslC* families, and finally by the *CslB*/*H*, *CslG*/*M*, and *CslE* genes. These groups respectively represent the oldest, the intermediate, and the most recent *CesA*/*Csl* families in evolutionary terms (Banasiak, 2014; Little et al., 2018). Moreover, we also found positive correlation between *CesA*/*Csl* copy number and whole genome duplications (WGDs) across species, suggesting that WGDs had a prominent role in driving *CesA*/*Csl* expansion and diversification. All together, these observations imply that novel duplicated *CesA*/*Csl* genes were typically retained by positive selection, contributing to expand, diversify, and sub-functionalize the *CesA* superfamily. Interestingly, Schwerdt et al. (2015) analysed nucleotide substitution rates of *CesA*/*Csl* genes from four grasses and found evidence of past positive selection for several of them followed by genomic stabilization, supporting our conclusion.

Since positive selection takes typically place when recently-duplicated genes provide adaptive advantages (Demuth and Hahn, 2009; Jensen and Bachtrog, 2010), it is relevant to investigate the advantages brought by novel *CesA/Csl* genes to plants. In this respect, we demonstrated that the *CesA* and *CslD* diversification accompanied sub-functionalization into primary and secondary *CesA* isoforms and different *CslD* functional groups (i.e., pollen development, determination of vegetative organ size, and root hair formation), respectively. Moreover, conserved phylogenomic patterns were found across all the main *CesA/Csl* families. We therefore hypothesize that the expansion and diversification of the *CesA* superfamily sustained the increased cell wall diversity during plant evolution, which in turn drove the evolution of land plants (Sarkar et al., 2009; Sørensen et al., 2010). In fact, cellulose and hemicelluloses have been fundamental for both the rise of land plants (by serving as sinks of photosynthesized carbohydrates and by providing mechanical support) and the rise of vascular and flowering plants (by modulating cell wall composition for vascularization, evolution of flowers and fruits, and pathogen resistance) (Sarkar et al., 2009). Remarkably, while all the major *CesA*/*Csl* families have members in basal land plants, the largest expansion of all the families was observed at the rise of flowering plants, fitting the evolutionary model just discussed.

The importance of *CesA*/*Csl* diversification in higher plants evolution was also highlighted by synteny data. Specifically, a consistent part of *CesA*/*Csl* syntenic communities is conserved across most angiosperms (Group C in Figure 2), suggesting genomic fixation of the successful diversification of *CesA*/*Csl* families into a highly conserved genomic background. This is remarkable, since the synteny of most angiosperm genes is typically lineage-specific (Zhao and Schranz, 2019). On the other hand, the fact that lineage-specific syntenic communities were nonetheless found (Groups A and B in Figure 2) demonstrates that *CesA*/*Csl* evolution is still dynamic and novel evolutionary trajectories take place on top, rather than in replacement of the common and stabilized genomic background. Lineage-specific gene arrangements may thus contribute to CW variability across plants. In this sense, the divergent genomic organization found in *CslA* genes between monocots and eudicots appears particularly marked, and we hypothesize that this could be at the basis of the profound differences in mannan and glucomannan content between these groups of plants (Vogel, 2008). This is further supported by the absence of clear differences between monocots and eudicots in terms of mere *CslA* copy number or gene sequence variability.

### Conserved Genomic Contexts in the CesA Superfamily: Biological Implications

A major finding of this research is the association between phylogenetic patterns, conservation (synteny) of such patterns, and gene sub-functionalization found in the *CesA* and *CslD* families. Moreover, similar patterns were detected in other *Csl* families, suggesting that such associations might also hold for other genes, but the absence of sufficient functional data hampered their detection. These observations question whether synteny is merely a trace of past evolutionary dynamics of *CesA*/*Csl* genes or if it can proactively contribute to determine gene function. In this respect, several elements support the second alternative. First, the exceptional extensiveness of *CesA*/*Csl* synteny compared to the typical lineage-specific synteny of angiosperm genes (Zhao and Schranz, 2019) suggests that selective constraints act against the positional genomic reshuffling of the *CesA* superfamily. In turn, this may indicate that synteny contributes to determine and conserve gene functions and adaptations, as hypothesized for other highly conserved gene families (Zhao et al., 2017; Kerstens et al., 2020). Furthermore, if this hypothesis is correct, positional constraints would likely be represented by tight mechanisms of gene regulation based on (epi)genetic properties variable along genomes and potentially disrupted by genome reshuffling (Gould et al., 2018; Crow et al., 2020; Lai et al., 2020). In this respect, several studies showed that cell wall biosynthesis is indeed hierarchically tightly regulated by the concerted action of complex plant gene networks (Taylor-Teeples et al., 2015; Rao and Dixon, 2018; Zhang et al., 2018). In addition, some *CesA* genes were shown to be targets of defined loops within such networks (Taylor-Teeples et al., 2015). Therefore, positional reshuffling of the *CesA*/*Csl* genes may significantly affect the deposition of cellulose and hemicellulosic polymers, which is strikingly what we found for the cotton secondary *CesA* genes transposed to conserved genomic contexts typical of primary *CesAs* in other plant species. All together, these observations support a central role for the *CesA* superfamily genomic architecture in determining gene function. However, given the fragmented evidence across the different *CesA*/*Csl* families – which is mostly due to the scarce functional characterization of several *Csl* genes – such roles should be better investigated in the future.

### The Evolution of the CesA Family

Based on the phylogenomic analyses, we proposed a novel evolutionary model for the *CesA* family, which hypothesizes that primary CW *CesAs* evolved before the secondary CW *CesAs* (Figure 5). These findings are in contrast with previous studies that claimed secondary *CesA* genes as the oldest ones by observing the primary CW *CesAs* phylogenetically nested within them (Schwerdt et al., 2015; Little et al., 2018). In our case, all the *CesA* phylogenetic trees (Figures 3, 5) positioned bryophyte and lycophyte *CesA* sequences within primary CW *CesA* branches (specifically clade 1 of Figures 3, 5), providing strong evidence in support of our model. Furthermore, the currently most-accepted framework of plant and cell wall evolution also agrees with our model. First, the detection of three groups of primary and three groups of secondary CesA isoforms only in gymnosperms and angiosperms agree with the pivotal role of secondary CW *CesAs* in the evolution of tall stems, wood, and vessels (Speck and Burgert, 2011; Cosgrove, 2012). Second, the fact that primary CWs alone sufficed the developmental needs of bryophytes and lycophytes (Sarkar et al., 2009) agrees with the finding of only primary *CesA* (-like) genes in these plants. Third, the intermediate evolutionary stage of ferns with a group of sequences clustered within clade 1 of Figure 5, another group preceding the split of the other two primary *CesA* clades, and a final group basal to the division of the three secondary CW *CesA* clades agree with the finding of *ancestral* conductive vessels in these species, with marked differences from the conductive vessels of angiosperms (Carlquist and Schneider, 2001; Sarkar et al., 2009). To conclude, the overall positioning of bryophyte/lycophyte, fern, and gymnosperm/angiosperm genes in the trees of Figures 3A, 5 suggests that the *CesA* family evolved over a long time, with the likely recurrent stabilization of its members into different intermediate diversified forms. Interestingly, this agrees with the most recent hypotheses on the evolution of the Cellulose Synthase Complexes (CSCs), which state that the hetero-oligomeric CSCs of higher plants (i.e. formed by different CesA isoforms) arose through constructive neutral evolution of ancestral homo-oligomeric complexes (CSCs of chlorophytes are formed by linear combination of interchangeable CesA proteins) pushed to differentiation by the diversification of their CesA subunits (Haigler and Roberts, 2019). While the sequence diversity of our bryophyte and lycophyte CesA sequences does not allow us to conclude whether they represent a single isoform, their positioning basal to the primary CW *CesA* branch in Figures 3A, 5 does not exclude this possibility. Therefore, the real morphology of CSCs in early-diverging land plants should be better elucidated in the future to answer the latter question. However, we think that the model of Haigler and Roberts (2019) overall agrees with the phylogenomic patterns observed in this research and further supports our hypotheses.

### The Evolutionary Relationship Between the CslD and CslF Families

The syntenic relationships between some *CslD* and *CslF* genes represent a last important finding of our research. Specifically, while the origin of the *CslF* family through the duplication of *CslD* members had been already reported based on phylogenetics (Yin et al., 2009; Schwerdt et al., 2015; Little et al., 2018), our results unveiled the genomic trace of this evolutionary link. This trace lays in the 1:2 syntenic connections found between a set of eudicot *CslD* members and both *CslD* and *CslF* genes from Poaceae. Since synteny involves both eudicot and grass *CslD* members, our results demonstrate that *CslF* genes originated when the *CslD* family was already formed, thanks to the relaxed selection pressure on duplicated grass *CslD* copies. Moreover, since synteny between *CslD* and *CslF* genes is found across all the Poaceae species in our analyses, the duplication at the origin of the *CslF* family may be shared by all the Poaceae. Therefore, assuming that a WGD event was responsible for this, such event could be the so-called ρ WGD, which is at the origin of the whole Poaceae lineage and took place about 56–70 Mya (Clark and Donoghue, 2018; Lee et al., 2020). To conclude, it remains unclear whether the *CslD* duplication described in this research originated all or only a part of the *CslF* family. In fact, the *CslF* genes syntenic to *CslD* members are the only ones genomically organized as singletons or in small tandem arrays, while no synteny between *CslF* and *CslD* genes was detected for the *CslF* members arranged into the large conserved *CslF* array (see section 2.1.2). Interestingly, such large conserved *CslF* array contains the two barley *CslF* genes that were recently found to synthesize a modified form of 1,4-linked polysaccharides termed (1,4)-β-linked glucoxylans (Little et al., 2019). Altogether, these observations indicate that (different parts of) the *CslF* family may have formed or expanded repeatedly during grass evolution, but the data produced in this research fail to highlight the specific modes of such evolution. Moreover, as for the (1,4)-β-linked glucoxylans of barley, these dynamic evolutionary patterns involving differential genomic arrangement of homologous genes may be at the basis of the formation of novel cell wall polysaccharides. However, the presence of this polysaccharide and of *CslF* genes responsible for its synthesis in species other than barley should be confirmed in the future to fully support the statement above. In conclusion, since the *CslF* genes syntenic with the *CslD* members are the phylogenetically oldest *CslFs* (Figure 6), we can confidently state that the *CslD* duplication was the first event in initiating the *CslF* family.

## Conclusion

In this research, we took advantage of novel bioinformatic tools for large-scale synteny, genomic, and phylogenetic analysis to improve our knowledge on the genetics, evolution, and functional characterization of the *CesA* superfamily. The tools we used – especially the combined analysis of synteny, phylogenetic, and functional data – provided the power to dissect the aspects just mentioned in the complex gene families at the basis of plant cell walls. However, the current level of functional characterization of many genes within the *CesA* superfamily is often hampering an effective use of the methodologies described here. Therefore, we foresee that progress in the functional characterization of cell wall genes through reverse genetics will be crucial to further advance our fundamental knowledge on these genes. In addition, future research can take advantage of our results to test the hypotheses we raise and close the open gaps in our understanding of the *CesA* superfamily.

## Data Availability Statement

The original contributions presented in the study are included in the article/Supplementary Material, further inquiries can be directed to the corresponding author.

## Author Contributions

FP designed and conducted this research and wrote the article, with inputs and supervision from LT, MS, and EL. LT, MS, and EL corrected the manuscript. LT, MS, and EL approved the final manuscript. All authors contributed to the article and approved the submitted version.

## Conflict of Interest

The authors declare that the research was conducted in the absence of any commercial or financial relationships that could be construed as a potential conflict of interest.

## Publisher’s Note

All claims expressed in this article are solely those of the authors and do not necessarily represent those of their affiliated organizations, or those of the publisher, the editors and the reviewers. Any product that may be evaluated in this article, or claim that may be made by its manufacturer, is not guaranteed or endorsed by the publisher.
