# Supplementary material for: Genomic Architecture and Evolution of the Cellulose synthase Gene Superfamily as Revealed by Phylogenomic Analysis
Source: Front Plant Sci. 2022 Apr 18;13:870818. doi: 10.3389/fpls.2022.870818 (PMC9062648; doi:10.3389/fpls.2022.870818)
Supplement: Supplementary file 1 [file Data_Sheet_1.PDF]

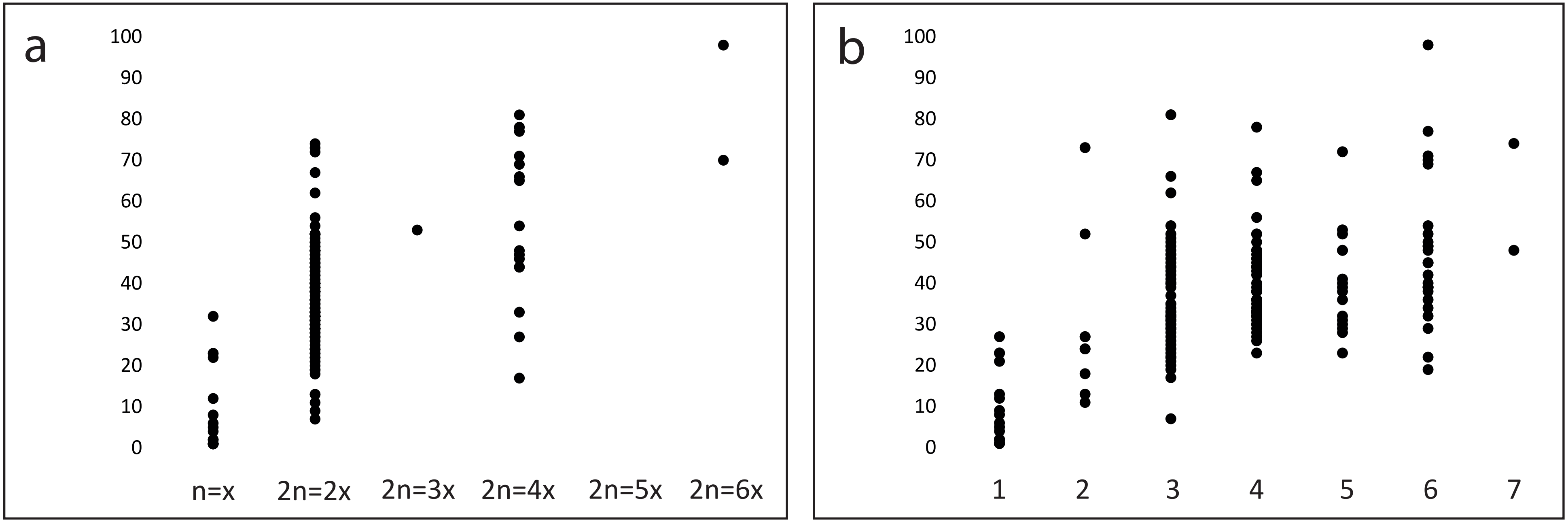

Supplementary Figure 1

a) Scatterplot of the ploidy level (x-axis) against the total number of CesA/Csl genes for the 222 plant genomes of the study with a BUSCO completeness of at least 75% (Viridiplantae set).

b) Scatterplot of the total number of WGDs to which a species has undergone along plant evolution against the total number of CesA/Csl genes for the 222 plant genomes of the study with a BUSCO completeness of at least 75% (Viridiplantae set). WGDs data were retrieved from Clark and Donoghue, 2018.

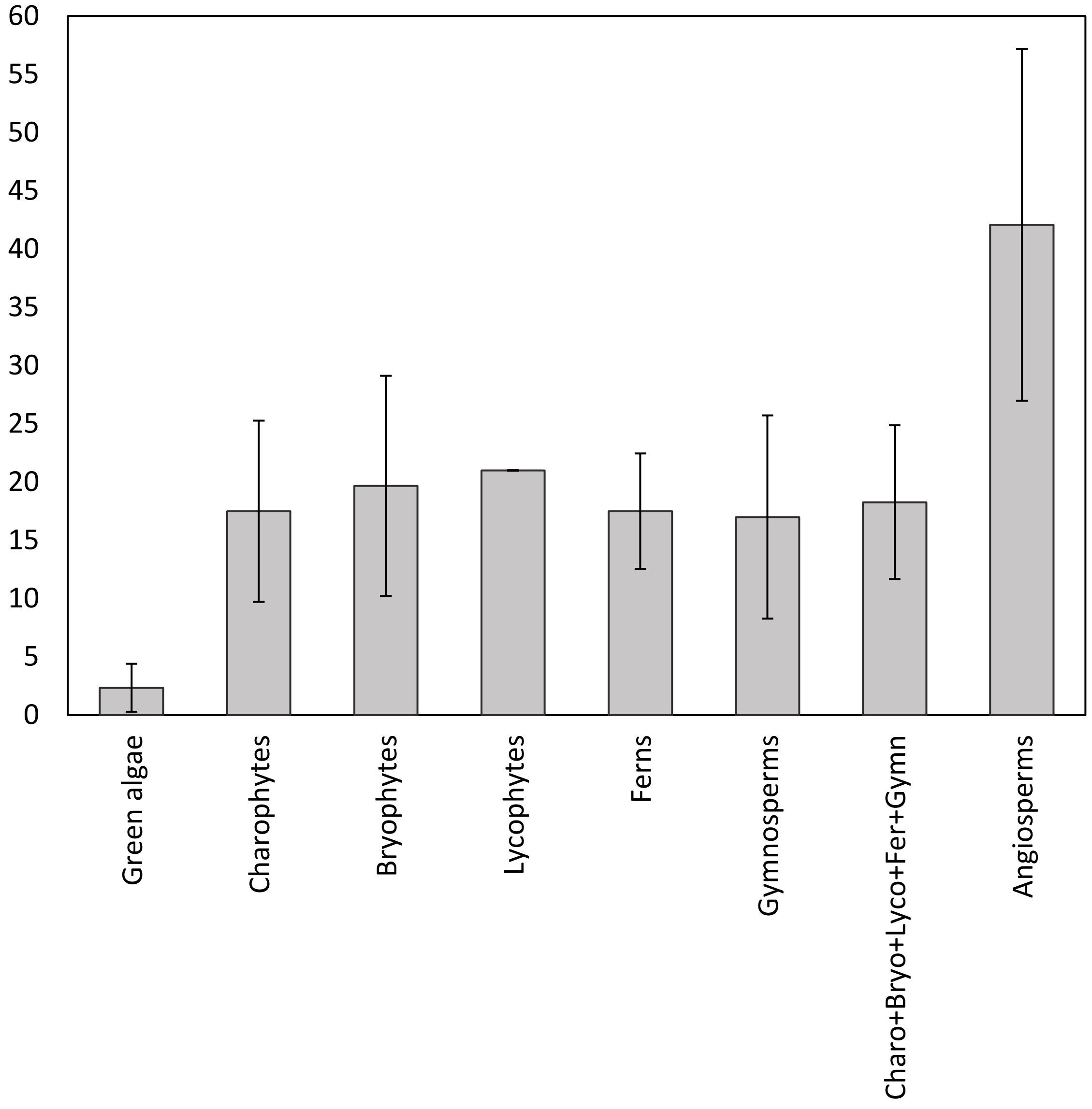

Supplementary Figure 2
Average number of CesA/Csl genes (y-axis) across main plant evolutionary clades (x-axis). Error bars represent the standard deviation of the mean. The data represented include the 222 plant genomes of the study with a BUSCO completeness of at least 75% (Viridiplantae set).

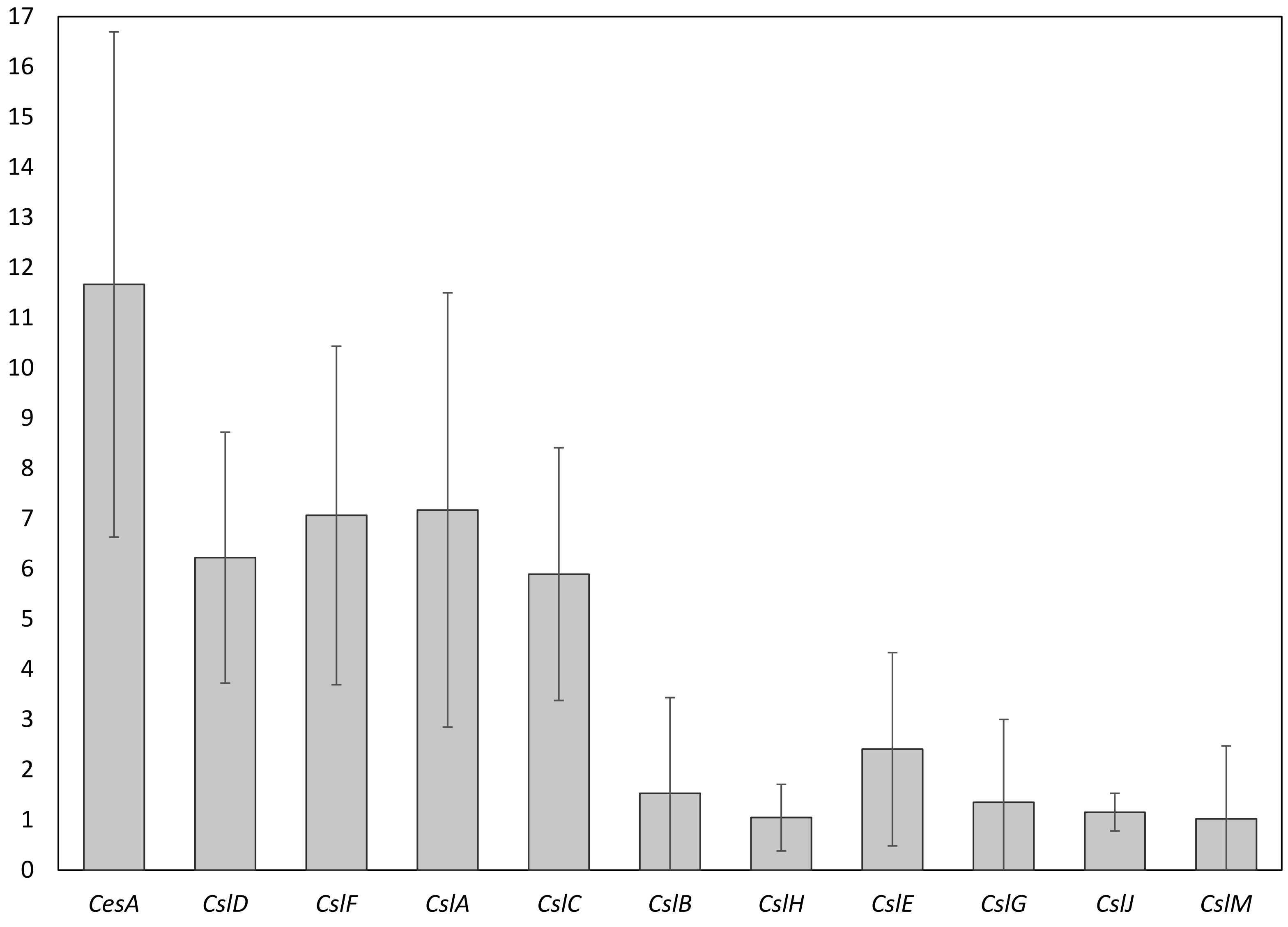

Supplementary Figure 3
Average number of genes across the 222 plant genomes of the study with a BUSCO completeness of at least 75% (Viridiplantae set; y-axis) for the 11 major gene families comprised within the CesA superfamily.
Error bars represent the standard deviation of the mean. Data for the green algae-specific CslK family are not shown.

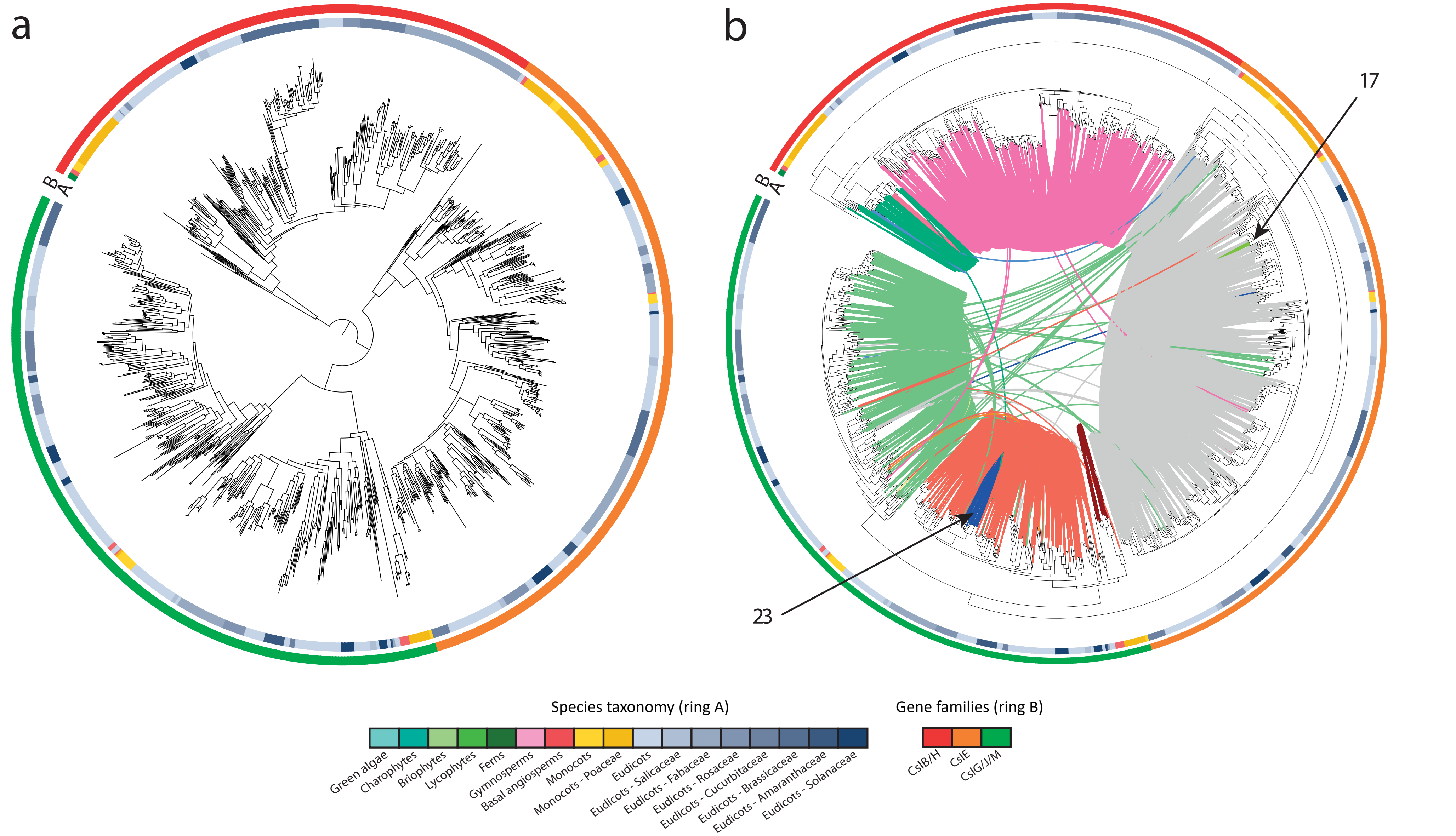

Supplementary Figure 4

a) The phylogenetic tree of the CslB/H, CslG/J/M and CslE families. Ring A indicates species taxonomy, while ring B indicates gene families (see legend).

b) The phylogenetic tree of the CslB/H, CslG/J/M and CslE families with the annotation of syntenic relationships between genes grouped by syntenic communities. Like in panel a, ring A indicates gene taxonomy, while ring B indicates gene families (see legend). The different colours of inner connections indicate distinct syntenic gene communities. Black arrows indicate syntenic communities 7 and 23, which display deviations from the commonalities observed between phylogenetic, syntenic, and functional patterns of these genes (see paragraph 3.2.4 in the article).

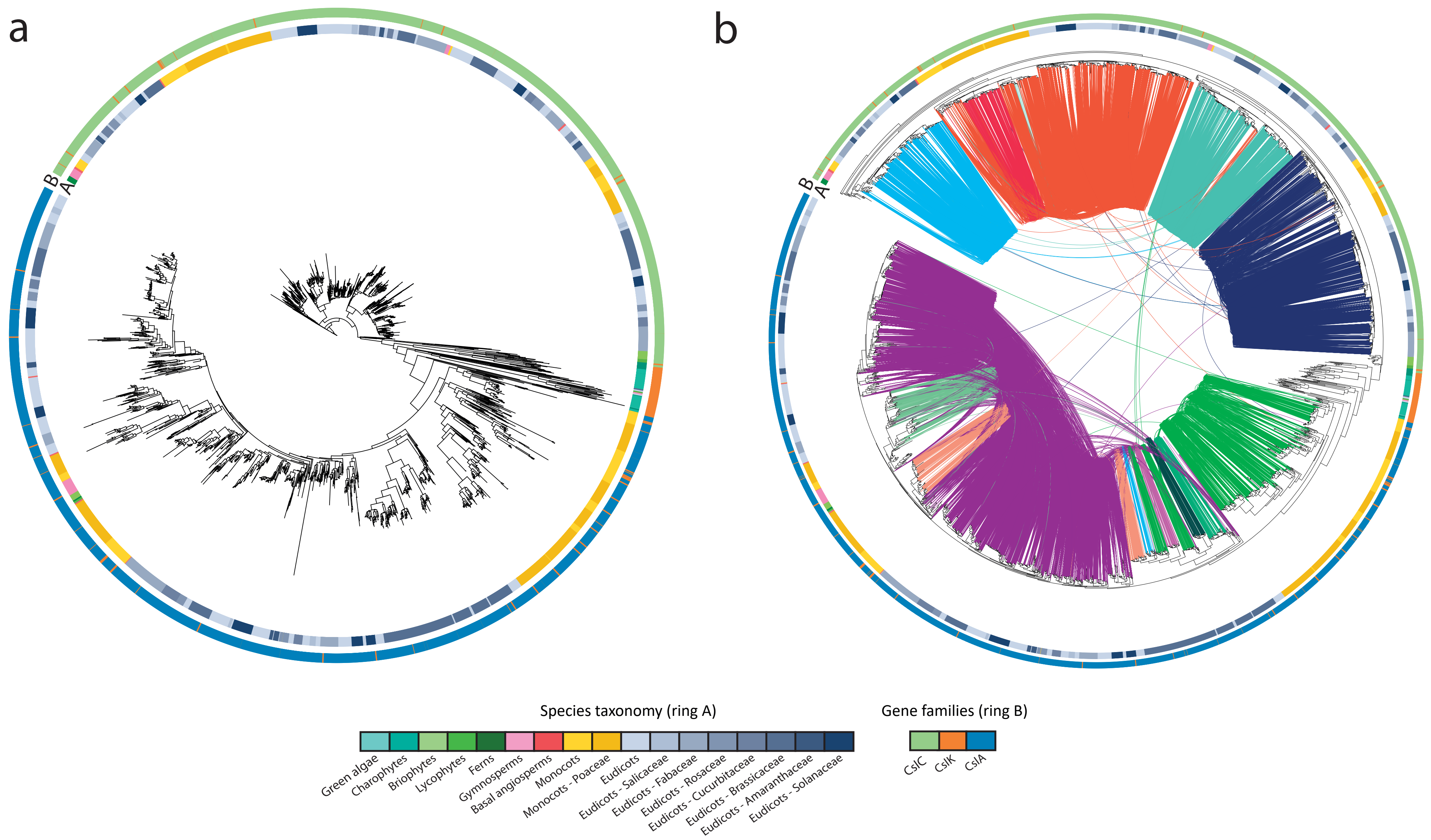

Supplementary Figure 5

a) The phylogenetic tree of the CslA/C/K families. Ring A indicates species taxonomy, while ring B indicates gene families (see legend). The orange bars within CslAs and CslCs areas indicate Csls that were not univoquely assigned to either CslA or CslC by BLAST and/or HMMER search (and therefore regarded as CslK genes). However, phylogeny places them within the CslA and CslC groups. "True" CslKs are represented by the group of orange Csl sequences on the right side of ring B.

b) The phylogenetic tree of the CslA/C/K families with the annotation of syntenic relationships between genes, grouped by syntenic gene communities. Like in panel a, ring A indicates species taxonomy, while ring B indicates gene families (see legend). The different colours of internal connections indicate distinct syntenic gene communities.
